# Potentially functional variants of *INPP5D and EXOSC3* in immunity B cell-related genes are associated with non-small cell lung cancer survival

**DOI:** 10.3389/fimmu.2024.1440454

**Published:** 2024-08-08

**Authors:** Guojun Lu, Hongliang Liu, Huilin Wang, Xiaozhun Tang, Sheng Luo, Mulong Du, David C. Christiani, Qingyi Wei

**Affiliations:** ^1^ Department of Respiratory Medicine, Nanjing Chest Hospital, Affiliated Nanjing Brain Hospital, Nanjing Medical University, Nanjing, China; ^2^ Duke Cancer Institute, Duke University Medical Center, Durham, NC, United States; ^3^ Department of Population Health Sciences, Duke University School of Medicine, Durham, NC, United States; ^4^ Department of Respiratory Oncology, Guangxi Cancer Hospital, Guangxi Medical University Cancer Hospital, Nanning, China; ^5^ Department of Head and Neck Surgery, Guangxi Cancer Hospital, Guangxi Medical University Cancer Hospital, Nanning, China; ^6^ Department of Biostatistics and Bioinformatics, Duke University School of Medicine, Durham, NC, United States; ^7^ Departments of Environmental Health and Epidemiology, Harvard TH Chan School of Public Health, Boston, MA, United States; ^8^ Department of Medicine, Massachusetts General Hospital, Boston, MA, United States; ^9^ Department of Medicine, Duke University Medical Center, Durham, NC, United States; ^10^ Duke Global Health Institute, Duke University Medical Center, Durham, NC, United States

**Keywords:** non-small cell lung cancer, immunity B cell, single-nucleotide polymorphism, overall survival, tumor microenvironment

## Abstract

B cells are adaptive immune cells in the tumor microenvironment and play an important role in tumor development and metastasis. However, the roles of genetic variants of the immunity B cell-related genes in the survival of patients with non-small cell lung cancer (NSCLC) remain unknown. In the present study, we first evaluated associations between 10,776 single nucleotide polymorphisms (SNPs) in 220 immunity B cell-related genes and survival of NSCLC in a discovery dataset of 1,185 patients from the Prostate, Lung, Colorectal and Ovarian (PLCO) Cancer Screening Trial. We found that 369 SNPs were significantly associated with overall survival (OS) of NSCLC in multivariable Cox proportional hazards regression analysis (*P* ≤ 0.05, Bayesian false discovery probability ≤ 0.80), of which 18 SNPs were validated in another independent genotyping dataset of 984 patients from the Harvard Lung Cancer Susceptibility (HLCS) Study. We then performed linkage disequilibrium (LD) analysis, followed by stepwise analysis with a multivariable Cox regression model. Finally, two independent SNPs, inositol polyphosphate-5-phosphatase D (*INPP5D*) rs13385922 C>T and exosome component 3 (*EXOSC3*) rs3208406 A>G, remained significantly associated withNSCLC OS with a combined hazards ratio (HR) of 1.14 (95% confidence interval = 1.06-1.23, *P* = 2.41×10^-4^) and 1.20 (95% confidence interval = 1.14-1.28, *P* = 3.41×10^-9^), respectively. Furthermore, NSCLC patients with the combination of unfavorable genotypes for these two SNPs were associated with a poor OS (*P*_trend_ = 0.0002) and disease-specific survival (DSS, *P*_trend_ < 0.0001) in the PLCO dataset. Expression quantitative trait loci (eQTL) analysis suggested that the *INPP5D* rs6782875 T allele was significantly correlated with elevated *INPP5D* mRNA expression levels in normal lung tissues and whole blood samples, while the *EXOSC3* rs3208406 G allele was significantly correlated with increased *EXOSC3* mRNA expression levels in normal lung tissues. Our data indicated that genetic variants in these immunity B cell-related genes may predict NSCLC survival possibly by influencing the gene expression.

## 1 Introduction

Lung cancer is the leading cause of cancer-related deaths in the world. In 2023, there were nearly 238,340 new cases diagnosed with and 127,070 deaths from lung cancer in the United States (1). In 2022 the National Cancer Center of China reported that lung cancer was both the most common cancer and the leading cause of cancer deaths in China (2). Therefore, lung cancer remains a substantial economic burden for both patients and healthcare systems globally (3). Based on its histological types, lung cancer can be divided into small cell lung cancer and non-small cell lung cancer (NSCLC), with the latter accounting for approximately 85% of all lung cancer cases (4). In recent years, despite remarkable advancements in earlier detection and therapeutic strategies, such as targeted molecular therapy and immunotherapy, the 5-year survival rate of advanced lung cancer remains low at only about 21% (5). However, individual lung cancer patients may respond dramatically differently to the same treatment and present different survival rates, and genetic variation may be involved in cancer progression (6). As a result, identifying genetic variation such as single-nucleotide polymorphisms (SNPs) in key genes and pathways may provide some new insights into the strategy of treating lung cancer.

The tumor microenvironment (TME) is a complex ecosystem where cancer cells are surrounded by diverse immune cells, inflammatory cells, tumor-associated fibroblasts, and altered extracellular matrix (7). Accumulating evidence suggests that TME has an important role in tumor initiation, progression, and metastasis (8, 9). As the second in the number of adaptive immune cells in TME, B cells localize to tumor-associated tertiary lymphoid structures and then interact with peripheral T cells and antigen-presenting cells to play a critical role in both pro-tumorigenic and anti-tumorigenic immunity (10). B cells not only promote tumor growth by secreting suppressive cytokines like IL-10, promoting immune tolerance by PD-L1^+^ B cells and producing proinflammatory cytokines such as IL-1β, but also inhibit tumor growth by secreting tumor-specific antibodies, serving as antigen-presenting cells themselves, and directly killing tumor cells (10–12). B cells were reported to be the second most common immune cell type with elevated expression levels in NSCLC tissues (13). A single-cell RNA-seq analysis indicated that plasma-like B cells inhibited tumor cell growth in the early stage and promoted cell growth in the advanced NSCLC (14). Moreover, the high percentage of naive‐like B cells in tumor tissues of NSCLC patients was associated with a better prognosis (14, 15). However, the potential role played by genetic variants in immunity B cell-related genes in NSCLC progression has not been reported.

Genome-wide association studies (GWASs) have been used to dissect the genotype-phenotype associations, providing new insights into the understanding of associations between genetic variants and certain diseases (16). For example, Chen et al. reported that genetic variants in peroxisome-related genes predicted NSCLC survival by influencing gene regulation (17). Another study indicated that two genetic variants in the immune-activation pathway genes affected the prognosis of NSCLC patients by regulating corresponding gene expression (18). In the present study, we have hypothesized that genetic variants in immunity B cell-related genes are associated with NSCLC survival. To test this hypothesis, we performed a two-stage analysis, using available GWAS data to evaluate associations between genetic variants in immunity B cell-related genes and survival of NSCLC patients.

## 2 Materials and methods

### 2.1 Study populations

In the two-stage analysis, we first used the GWAS dataset of lung cancer patients of European ancestry from the Prostate, Lung, Colorectal, and Ovarian cancer screening trial (PLCO) Cancer Screening Trial as the discovery. The PLCO enrolled approximately 155,000 participants aged 55-74 from 10 competitively selected screening centers across the United States between 1993 and 2001. Among all the participants, we extracted 1185 NSCLC patients (487 women and 698 men) with detailed personal information such as age, sex, smoking status, treatment history, and follow-up time for further survival analysis. Genotyping data were extracted from whole blood DNA samples genotyped using Illumina HumanHap240Sv1.0 and HumanHap550v3.0 platforms (dbGaP accession numbers: phs000093.v2.P2 and phs000336.v1.p1) (19, 20). The PLCO trial was approved by the National Cancer Institute and the institutional review boards of each involved center.

Then, we used another genotyping dataset from the Harvard University Lung Cancer Susceptibility (HLCS) study to validate the findings of the PLCO dataset. HLCS study included 984 histologically confirmed Caucasian NSCLC patients from the Massachusetts General Hospital (MGH) (21). The genomic blood DNA samples were used for genotyping by the Illumina Humanhap610-Quad array, and the genotyping data were subsequently imputed with the software MaCH based on the 1000 Genomes Project.

The use of the PLCO trial and HLCS study for experimentation was approved by the Internal Review Board of Duke University School of Medicine (Project #Pro00054575) and the dbGaP database (Project #6404). The characteristics of the two datasets are shown in **Supplementary Table S1**.

### 2.2 Gene selection and SNP imputation

We searched for the immunity B cell-related genes by using the keywords “B cell” and “immunity” from the Molecular Signature Database (http://www.gsea-msigdb.org/gsea/msigdb/human/search.jsp) (22). After excluding duplicated genes and genes on the X chromosome, 220 remaining genes were identified as candidate genes for further analyses (**Supplementary Table S2**). SNPs with ±2kb flanking regions of 220 immunity B cell-related genes were extracted from the PLCO trial and conducted using the Minimac4 based on the European data in the 1000 Genomes Project (phase 3). For the control quality, all the SNPs were extracted according to the following criteria: an imputation info score ≥ 0.3 (**Supplementary Figure S1**), a minor allele frequency (MAF) ≥ 5%, an individual call rate ≥ 95%, and the Hardy-Weinberg equilibrium *P*-value (HWE) ≥ 1 × 10^−5^. Finally, a total of 10,776 SNPs (1,196 genotyped and 9,580 imputed) were obtained from the PLCO dataset for further analysis.

### 2.3 Statistical methods

In the PLCO dataset, to estimate the associations between 10,776 candidate SNPs and NSCLC survival in an additive genetic model, we first performed single-locus Cox proportional hazards regression analysis using the R package GenABEL package (23). The Cox regression analysis was performed with adjustment for clinical variables (including age, sex, smoking status, histologic subtype, tumor stage, chemotherapy, radiotherapy, and surgery) and the top four of the 10 principal components (PCs) in the discovery dataset (**Supplementary Table S3**). To filter out potential false-positive results, we employed a multiple testing correction by Bayesian false discovery probability (BFDP) with the threshold of 0.8 as recommended (24). We used a prior probability of 0.10 to detect an upper boundary hazards ratio (HR) of 3.0 for an association with variant genotypes or minor alleles of the SNPs with *P* < 0.05.

The identified SNPs from the PLCO GWAS dataset were validated using the GWAS dataset of the HLCS study in a multivariable Cox regression model. To combine the results of the two GWAS datasets, the inverse variance weighted meta-analysis was performed, using Cochran’s Q-test and the heterogeneity statistic (*I^2^*) to assess inter-study and determine the appropriate model. If no heterogeneity (Q-test *P* > 0.10 and *I* ^2^ < 50%), the meta-analysis was performed with a fixed-effects model, otherwise with the random-effects model.

Linkage disequilibrium (LD) analysis was performed with Haploview 4.1. Two online bioinformatics tools, RegulomeDB (http://www.regulomedb.org/) and HaploReg v4.2 (https://pubs.broadinstitute.org/mammals/haploreg/haploreg.php), were used to predict the potentially functional SNPs (25, 26). Subsequently, to identify the associations between independent SNPs and NSCLC survival, we employed the multivariable stepwise Cox regression model with adjustment for demographic and clinical variables, the top four PCs, and 54 previously published SNPs from the same PLCO GWAS dataset. We also generated the Manhattan plots with Haploview4.1 and regional association plots with Locus Zoom (http://http://locuszoom.sph.umich.edu) to visualize the selected SNPs (27).

Subsequently, we employed the combined unfavorable genotypes to evaluate the cumulative effects of the two identified SNPs and the Kaplan-Meier (KM) survival curves to assess the survival probability. We also performed stratified analysis and evaluated inter-study heterogeneity to assess the associations between subgroups and survival as well as the combined effect of unfavorable genotypes that might be influenced by clinical characteristics. To evaluate predictive accuracy of the clinical models with the addition of the genetic variables, we constructed time-dependent area under the curve (AUC) and the receiver operating characteristic (ROC) curves using R (version 3.6.3) package “time ROC” and “survival” (28).

To explore the genotype-phenotype correlation between two identified SNPs and mRNA expression levels of their corresponding genes, we performed expression quantitative trait loci (eQTL) analyses with a linear regression model using data from two sources: lymphoblastoid cell lines in 373 European descendants from the 1,000 Genomes Project (Phase 3) and the genotype-tissue expression (GTEx) project (including 515 normal lung tissues and 670 whole blood samples) (29). To compare the mRNA expression among tumor tissue of lung adenocarcinoma (LUAD), lung squamous cell carcinoma (LUSC) and adjacent normal tissue with paired and unpaired *t*-tests, we downloaded the raw expression data from the Cancer Genome Atlas (TCGA) database and performed the analyses on the online data platform UALCAN (https://ualcan.path.uab.edu/) (30), respectively. Finally, we assessed the correlations between the mRNA expression levels of two genes and NSCLC survival probability using the online survival analysis database Kaplan-Meier (http://kmplot.com/analysis/) (31). Unless specified otherwise, all statistical analyses were conducted with the SAS software 9.4 (SAS Institute, Cary, NC, USA).

## 3 Results

### 3.1 Associations between SNPs in the immunity B cell-related genes and the survival of NSCLC

**Figure 1:**
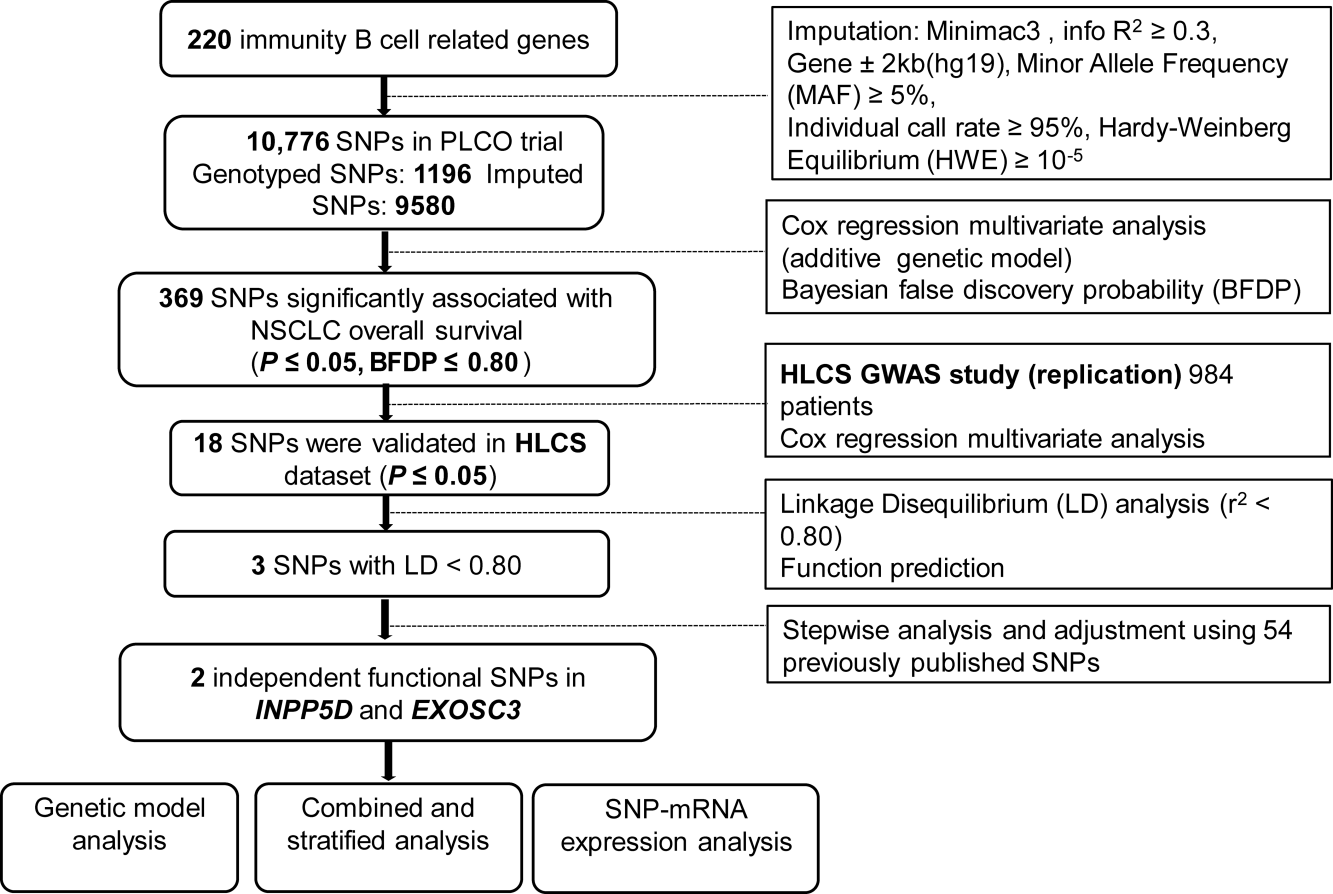
The flowchart of the present study. SNP, single-nucleotide polymorphism; PLCO, Prostate, Lung, Colorectal and Ovarian cancer screening trial; NSCLC, non-small cell lung cancer; HLCS, Harvard lung cancer susceptibility study; GWAS, Genome-Wide Association Study.

The final analysis in the present study included 1185 NSCLC patients from the PLCO trial and 984 NSCLC patients from the HLCS study, and their clinical features are described in **Supplementary Table S1**. The study flow chart is depicted in **Figure 1**. After multiple testing corrections by BFDP (≤ 0.80), 369 SNPs out of the 10,776 SNPs in the immunity B cell-related genes were found to be statistically significantly associated with NSCLC overall survival (OS) (*P* ≤ 0.05); then, these SNPs were further validated in the HLCS dataset. As a result, 18 SNPs in three genes (i.e., inositol polyphosphate-5-phosphatase D, *INPP5D*; complement factor I, *CFI*; and exosome component 3, *EXOSC3*) remained significant. While *EXOSC3* has only one SNP (i.e., rs3208406 in *EXOSC3*), further LD analyses of the remaining 17 SNPs with Haploview 4.1 software identified two SNPs (one in each of *INPP5D* and *CFI*) as the tagger SNPs (**Supplementary Figure S2**). The results of subsequent online functional prediction for these three SNPs are listed in **Supplementary Table S4**. *EXOSC3* rs3208406 A>G and *CFI* rs6836770 G>A may have an effect on enhancer histone marks, and the allele change in these three SNPs may alter protein motifs.

### 3.2 Identification the effect of independent SNPs on NSCLC OS in the PLCO trial

Because the HLCS replication dataset did not have the same genotyping data as the PLCO did, to identify the effect of independent SNPs on NSCLC OS, we first used the PLCO genotyping dataset to perform the stepwise multivariable Cox regression analysis. Next, we put the remaining significant SNPs into a post-stepwise multivariable model including 54 previously reported SNPs in the PLCO GWAS dataset. Finally, two SNPs (*INPP5D* rs13385922 C>T and *EXOSC3* rs3208406 A>G) remained significantly associated with NSCLC OS (*P* = 0.003 and 0.002, respectively) (**Table 1**). Moreover, the meta-analysis of the PLCO trial and HLCS study revealed that there was no interstudy heterogeneity across these two datasets, and the combined results are listed in **Table 2**. We also depicted Manhattan plots (**Supplementary Figure S3**) and regional association plots (**Supplementary Figure S4**) for these two significant SNPs.

**Table 1:** Two independent SNPs in multivariable Cox proportional hazards regression analysis with adjustment for other covariates and 54 previously published SNPs in the PLCO dataset.

Variables	Category	Frequency	HR (95% CI) ^a^	*P* ^a^	HR (95% CI) ^b^	*P* ^b^
Age	Continuous	1185	1.03 (1.02-1.04)	<0.0001	1.05 (1.03-1.06)	<0.0001
Sex	Male	698	1.00		1.00	
	Female	487	0.81 (0.69-0.94)	0.006	0.65 (0.55-0.78)	<0.0001
Smoking status	Never	115	1.00		1.00	
	Current	423	1.67 (1.24-2.25)	0.0006	2.31 (1.67-3.19)	<0.0001
	Former	647	1.65 (1.25-2.18)	0.0004	2.26 (1.67-3.07)	<0.0001
Histology	AD	577	1.00		1.00	
	SC	285	1.14 (0.95-1.38)	0.166	1.13 (0.92-1.39)	0.246
	Others	323	1.31 (1.10-1.56)	0.002	1.46 (1.21-1.77)	0.0001
Stage	I-IIIA	655	1.00		1.00	
	IIIB-IV	528	2.76 (2.26-3.36)	<0.0001	3.98 (3.18-4.97)	<0.0001
Chemotherapy	No	639	1.00		1.00	
	Yes	538	0.59 (0.49-0.70)	<0.0001	0.47 (0.38-0.57)	<0.0001
Radiotherapy	No	762	1.00		1.00	
	Yes	415	0.93 (0.79-1.10)	0.417	1.10 (0.92-1.32)	0.291
Surgery	No	637	1.00		1.00	
	Yes	540	0.20 (0.16-0.26)	<0.0001	1.63 (1.23-2.15)	<0.0001
*INPP5D* rs13385922 C>T	CC/CT/TT	432/560/192	1.17 (1.06-1.30)	0.002	1.18 (1.06-1.32)	0.003
*EXOSC3* rs3208406 A>G	AA/AG/GG	979/172/7	1.24 (1.04-1.49)	0.019	1.39 (1.13-1.71)	0.002

HR, hazards ratio; CI, confidence interval; SNP, single-nucleotide polymorphisms; PLCO, Prostate, Lung, Colorectal and Ovarian cancer screening trial; INPP5D, Inositol polyphosphate-5-phosphatase.

a Stepwise analysis included age, sex, smoking status, tumor stage, histology, chemotherapy, radiotherapy, surgery, PC1, PC2, PC3, PC4 and SNPs.

b 54 published SNPs were used for post-stepwise adjustment: rs779901, rs3806116, rs199731120, rs10794069, rs1732793, rs225390, rs3788142, rs73049469, rs35970494, rs225388,rs7553295, rs1279590, rs73534533, rs677844, rs4978754, rs1555195, rs11660748, rs73440898, rs13040574, rs469783, rs36071574, rs7242481, rs1049493,rs1801701, rs35859010, rs1833970, rs254315, rs425904, rs35385129, rs4487030,rs60571065, rs13213007, rs115613985, rs9673682, rs2011404, rs7867814, rs2547235,rs4733124, rs11225211, rs11787670, rs67715745, rs922782, rs4150236, rs116454384, rs9384742, rs9825224, rs261083, rs76744140, rs6939623, rs113181986, rs2568847, rs10841847, rs2519996, rs36215.

**Table 2:** Associations of two independent SNPs with overall survival in both discovery and validation datasets from two previously published NSCLC GWASs.

SNP	Allele ^a^	Gene	PLCO (n=1185)					Harvard (n=984)		Meta-analysis			
			BFDP	EAF	HR (95%CI) ^b^	*P* ^b^	EAF	HR (95%CI) ^c^	*P* ^c^	HR (95%CI) ^d^	*P* ^d^	*P*_het_ ^e^	I ^2^
rs13385922	C>T	*INPP5D*	0.28	0.40	1.18 (1.06-1.30)	0.002	0.42	1.11 (1.00-1.22)	0.050	1.14 (1.06-1.23)	2.41×10^-4^	0.376	0
rs3208406	A>G	*EXOSC3*	0.77	0.08	1.25 (1.04-1.50)	0.016	0.10	1.20 (1.02-1.41)	0.031	1.20 (1.14-1.28)	3.41×10^-9^	0.669	0

EAF, effect allele frequency; HR, hazards ratio; CI, confidence interval.

a Reference>effect allele.

b Adjusted for age, sex, stage, histology, smoking status, chemotherapy, radiotherapy, surgery, PC1, PC2, PC3 and PC4.

c Adjusted for age, sex, stage, histology, smoking status, chemotherapy, radiotherapy, surgery, PC1, PC2, and PC3.

d Meta-analysis in the fix-effects model.

e P_het_, P value for heterogeneity by Cochrane’s Q test.

As shown in **Table 3**, both *the INPP5D* rs13385922 *T* allele and *EXOSC3* rs3208406 G allele were significantly associated with OS (*P*_trend_ = 0.003 and 0.023, respectively) and disease-specific survival (DSS) (*P*_trend_ = 0.0005 and 0.003, respectively). Compared with those having the reference genotype in a dominant genetic model, NSCLC patients had a significantly poor survival associated with *INPP5D* rs13385922 CT+TT (OS: HR = 1.22, 95% CI = 1.06-1.42, and *P* = 0.008; DSS: HR = 1.29, 95% CI = 1.10-1.51, and *P* = 0.002), and with *EXOSC3* rs3208406 AG+GG (OS: HR = 1.27, 95% CI = 1.05-1.55, and *P* = 0.015; DSS: HR = 1.37, 95% CI = 1.12-1.67, and *P* = 0.002). Additionally, we also depicted Kaplan-Meier survival curves for these results (**Supplementary Figure S5**).

**Table 3:** Associations between two independent SNPs and survival of NSCLC in the PLCO trial.

Genotype	Frequency	OS ^a^			DSS ^a^		
		Death (%)	HR (95% CI)	*P*	Death (%)	HR (95% CI)	*P*
*INPP5D* rs13385922 C>T ^b^							
CC	428	277 (64.72)	1.00		239 (55.84)	1.00	
CT	554	372 (67.15)	1.18 (1.01-1.39)	0.038	337 (60.83)	1.24 (1.05-1.47)	0.013
TT	192	139 (72.40)	1.36 (1.10-1.67)	0.004	132 (68.75)	1.44 (1.16-1.79)	0.001
Trend test				0.003			0.0005
Dominant							
CC	428	277 (64.72)	1.00		239 (55.84)	1.00	
**CT+TT**	**746**	**511 (68.50)**	**1.22 (1.06-1.42)**	**0.008**	**469 (62.87)**	**1.29 (1.10-1.51)**	**0.002**
*EXOSC3* rs3208406 A>G ^c^							
AA	970	649 (66.91)	1.00		575 (59.28)	1.00	
AG	171	119 (69.59)	1.29 (1.06-1.57)	0.013	113 (66.08)	1.37 (1.12-1.69)	0.002
GG	7	4 (57.14)	1.00 (0.37-2.67)	0.992	4 (57.14)	1.16 (0.43-3.12)	0.771
Trend test				0.023			0.003
Dominant							
AA	970	649 (66.91)	1.00		575 (59.28)	1.00	
**AG+GG**	**178**	**123 (69.10)**	**1.27 (1.05-1.55)**	**0.015**	**117 (65.73)**	**1.37 (1.12-1.67)**	**0.002**
NUG ^d,e^							
0	350	220 (62.86)	1.00		183 (52.29)	1.00	
1	688	481 (69.91)	1.32 (1.12-1.56)	0.0009	443 (64.39)	1.43 (1.20-1.71)	<0.0001
2	109	70 (64.22)	1.50 (1.14-1.97)	0.003	65 (59.63)	1.67 (1.25-2.22)	0.0005
Trend test				0.0002			<0.0001
0	350	220 (62.86)	1.00		183 (52.29)	1.00	
1-2	797	551 (69.13)	1.34 (1.14-1.58)	0.0003	508 (63.74)	1.46 (1.23-1.73)	<0.0001

SNP, single nucleotide polymorphism; NSCLC, non-small cell lung cancer; PLCO, Prostate, Lung, Colorectal and Ovarian cancer screening trial; OS, overall survival; DSS, disease-specific survival; HR, hazards ratio; CI, confidence interval.

a Adjusted for age, sex, smoking status, histology, tumor stage, chemotherapy, surgery, radiotherapy and top four principal components.

b 11 missing data were excluded.

c 37 missing data were excluded.

d 38 missing data were excluded.

e Unfavorable genotypes were INPP5D rs13385922 CT+TT and EXOSC3 rs3208406 AG+GG and their results are in bold.

### 3.3 Combined effect of two independent SNPs on NSCLC survival in the PLCO dataset

**Figure 2:**
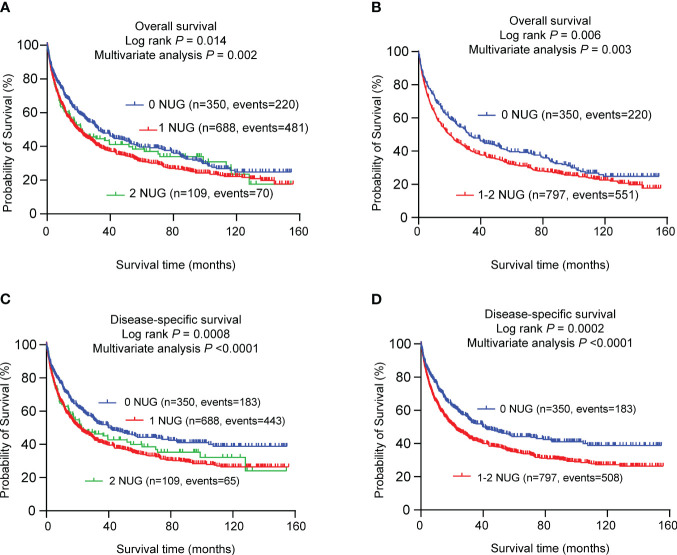
Prediction of survival with the combined unfavorable genotypes. Kaplan–Meier survival curves in the PLCO dataset for **(A)** OS with the combined unfavorable genotypes; **(B)** OS with the dichotomized groups of the NUGs; **(C)** DSS with the combined unfavorable genotypes; **(D)** DSS with dichotomized groups of the NUGs. ^#^Unfavorable genotypes were *INPP5D* rs13385922 CT+TT and *EXOSC3* rs3208406 AG+GG. SNPs, single nucleotide polymorphism; NUG, number of unfavorable genotypes; PLCO, The Prostate, Lung, Colorectal and Ovarian Cancer Screening Trial.

To assess the combined effect of these two independent SNPs on NSCLC survival, we combined unfavorable genotypes (*INPP5D* rs13385922 CT+TT and *EXOSC3* rs3208406 AG+GG) into a genetic score and divided all NSCLC patients into three groups (i.e., 0, 1, and 2) according to the number of unfavorable genotypes (NUGs). As shown in **Table 3**, an elevated NUG score was associated with a poor survival for both OS (*P*_trend_ < 0.0002) and DSS (*P*_trend_ < 0.0001). Furthermore, we dichotomized the NUGs and divided all NSCLC patients into low-unfavorable-genotypes group (0 NUGs) and high-unfavorable-genotypes group (1-2 NUGs). Compared with the 0 NUGs group, the 1-2 NUGs group had a significantly poorer survival for OS (HR = 1.34, 95% CI = 1.14-1.58, and *P* = 0.0003) and DSS (HR = 1.46, 95% CI = 1.23-1.73, and *P*_trend_ < 0.0001). We also depicted these results with Kaplan-Meier survival curves from a log-rank perspective (**Figures 2A–D**).

### 3.4 Stratified analysis for associations between NUGs and NSCLC survival

To assess the possible modification effect of NUGs on NSCLC survival by age, sex, smoking status, histology, tumor stage, chemotherapy, radiotherapy, and surgery, we further conducted stratified analysis in the PLCO trial. As shown in **Supplementary Table S5**, For the effects of both 0 and 1-2 NUG groups on NSCLC OS and DSS, no significant interactions were found between NUGs and age, sex, smoking status, histology, tumor stage, chemotherapy, radiotherapy, and surgery (all *P*_inter_ > 0.05).

### 3.5 Time-dependent AUC and ROC curves to predict NSCLC survival

To further evaluate the predictive role in survival of the two SNPs for OS and DSS, we performed the time-dependent AUC and ROC curves at the 12^th^,36^th^, and 60^th^ month in the PLCO trial. Compared with the predictive model for clinical covariates including age, sex, smoking status, histology, tumor stage, chemotherapy, radiotherapy, surgery, and the four PCs, the predictive values before and after adding the two independent SNPs to the model were different. Time-dependent AUC for OS and DSS were shown in **Supplementary Figures S6A, B**. With the addition of the two SNPs, the AUC increased from 87.38% to 88.08% for OS (*P* = 0.020) and from 87.48% to 88.20% for DSS (*P* = 0.039) at 12^th^ month (**Supplementary Figures S6C, D**). However, the predictive performance of AUC curves at the 36^th^ and 60^th^ month for both OS and DSS was not significantly improved (all *P* > 0.05, **Supplementary Figures S6E–H**).

### 3.6 The result of eQTL analysis

**Figure 3:**
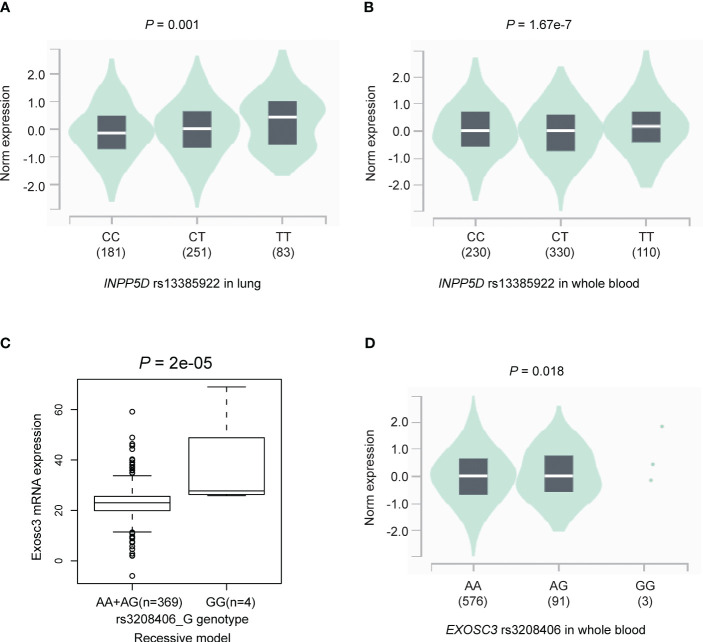
The results of the eQTL analysis. The *INPP5D* rs13385922 T allele was significantly associated with high mRNA expression levels of *INPP5D* in **(A)** normal lung tissues and **(B)** whole blood samples in the GTEx project; the *EXOSC3* rs3208406 G allele was associated with high mRNA expression levels of *EXOSC3* in **(C)** recessive model in the lymphoblastoid cell lines from the 1000 Genomes Project and **(D)** whole blood samples in the GTEx project. eQTL, expression quantitative trait loci.

To explore the genotype-phenotype correlation, we first performed the eQTL analysis using genomic data of lymphoblastoid cell lines from the 373 European descendants in the 1000 Genomes Project. The results suggested that the *INPP5D* rs13385922 T allele was not associated with expression levels of *INPP5D* mRNA in the additive (*P* = 0.405, **Supplementary Figure S7A**), dominant model (*P* = 0.430, **Supplementary Figure S7B**), and recessive model (*P* = 0.586, **Supplementary Figure S7C**). We also employed an eQTL analysis using GTEx project data. The results suggested that the *INPP5D* rs13385922 T allele was significantly associated with high mRNA expression levels of *INPP5D* in both normal lung tissues (*P* = 0.001, **Figure 3A**) and whole blood samples (*P* = 1.67e-7, **Figure 3B**). Moreover, the *EXOSC3* rs3208406 G allele was associated with high mRNA expression levels of *EXOSC3* in the recessive model (*P* = 2e-05, **Figure 3C**), but not in the additive (*P* = 0.117, **Supplementary Figure S7D**) and dominant model (*P* = 0.557, **Supplementary Figure S7E**). In the GTEx project, the *EXOSC3* rs3208406 G allele was associated with mRNA expression levels of *EXOSC3* whole blood samples (*P* = 0.018, **Figure 3D**), but not in normal lung tissues (*P* = 0.105, **Supplementary Figure S7F**).

### 3.7 The analysis of mRNA expression and survival in NSCLC

**Figure 4:**
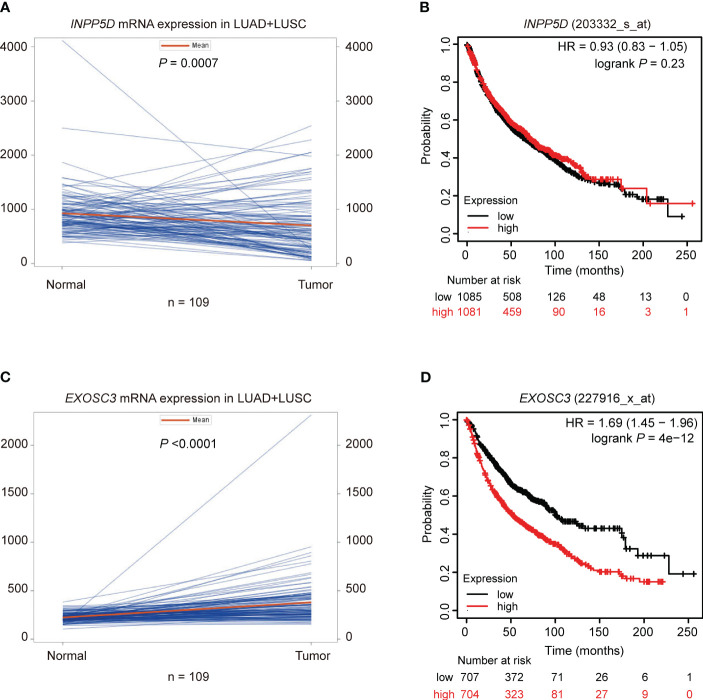
Paired mRNA expression and survival analyses in NSCLC. Paired *t*-test suggested that the mRNA expression of **(A)** *INPP5D* was down-regulated and **(C)** *EXOSC3* was up-regulated in NSCLC; survival analysis suggested that **(B)** mRNA expression levels of *INPP5D* were not associated with NSCLC survival; **(D)** high mRNA expression levels of *EXOSC3* were associated with a poor NSCLC survival.

To explore potential mechanisms of *INPP5D* and *EXOSC3* on NSCLC survival, we first evaluated the mRNA expression levels of these two genes using paired *t*-tests with data from the TCGA database and unpaired tests with the online UALCAN portal. Then, we used Kaplan-Meier Plotter web tool to estimate the associations between their mRNA expression levels and NSCLC survival. Compared with adjacent paired normal tissues, *INPP5D* mRNA expression was significantly down-regulated in tissues from the combined LUSC and LUAD (*P =* 0.0007) (**Figure 4A**), and LUSC (*P <* 0.0001), but not from LUAD (*P =* 0.565) (**Supplementary Figures S8A, B**). Similar results were also observed for LUSC (*P <* 0.0001) and LUAD (*P =* 0.888) in the UALCAN database using unpaired tests (**Supplementary Figures S8C, D**). Furthermore, mRNA expression levels of *INPP5D* were not associated with OS of NSCLC (HR = 0.93, 95% CI: 0.83-1.05, log-rank *P* = 0.23) (**Figure 4B**). *EXOSC3* mRNA expression levels were significantly up-regulated in tissues from the combined LUSC + LUAD (**Figure 4C**), LUSC, and LUAD (**Supplementary Figures S8E, F**) (all *P <* 0.0001) than those in adjacent normal tissues using paired *t*-test. Similar results were also observed for LUSC and LUAD (all *P <* 0.0001) in the UALCAN database using unpaired tests (**Supplementary Figures S8G, H**). Moreover, high mRNA expression levels of *EXOSC3* were associated with a poor NSCLC OS (HR = 1.69, 95% CI: 1.45-1.96, log-rank *P* = 4e-12) (**Figure 4D**).

## 4 Discussion

In the present study, we evaluated the associations between 10,776 genetic variants in the 220 immunity B cell-related genes and NSCLC survival using available GWAS genotyping data from both PLCO trial and HLCS study. Our results indicated that the *INPP5D* rs13385922 C>T and *EXOSC3* rs3208406 A>G were significantly associated with a poor survival in the United States Caucasian populations. Our findings also suggested that the *INPP5D* rs13385922 variant T allele and *EXOSC3* rs3208406 variant G allele were associated with up-regulated mRNA expression levels of *INPP5D* and *EXOSC3* in the lymphoblastoid cell lines from the 1000 genomes project and the GTEx project, implying that these alleles may modulate mRNA expression and influence NSCLC survival. Our findings provided additional support for associations between genetic variants in the immunity B cell-related genes and survival of NSCLC patients.

B cells are the second most numerous adaptive immune cells in TME and may mediate both pro- and antitumorigenic effects in tumor development (32, 33). Within the tertiary lymphoid structures, B cells perform the function of antigen presentation and antibody production to focus immune responses, and the expression of B cells is associated with clinical outcomes in multiple cancer types (34, 35). However, up to now no studies have reported the association between functional genetic variants of immunity B cell-related genes and NSCLC survival. To the best of our knowledge, this is the first study to explore the role of genetic variants of immunity B cell-related genes in predicting NSCLC survival. As a result, we identified two SNPs (i.e., *INPP5D* rs13385922 C>T and *EXOSC3* rs3208406 A>G) from immunity B cell-related genes that predicted the prognosis of NSCLC patients.

*INPP5D*, inositol polyphosphate-5-phosphatase D (all known as SHIP1), is located on chromosome 2 and composed of 1189 amino acids. *INPP5D* is a member of the inositol polyphosphate-5-phosphatase (*INPP5*) family and plays an important role in the immune system (36). A previous study reported that *INPP5D* can skew macrophage progenitors toward M1 macrophages and naive T cells to T helper 1 and T helper 17 cells; as a result, *INPP5D* is intricately linked to the activation of the immune system and plays a key role in the solid tumor eradication (37). Pulsatile *INPP5D* inhibition contributed to the enhancement of T and NK cell function and improved antitumor immunity and survival in mouse models of lymphoma and colon cancer (38). *INPP5D* has been shown to suppress the activity of PI3K/AKT/mTOR signaling pathway via reducing PI(3,4,5)P3 levels at the plasma membrane and promote cancer cell survival (39). In NSCLC, the expression of *INPP5D* was down-regulated in both tumor tissues and cell lines, and the overexpression of *INPP5D* suppressed cell growth, migration, and invasion by inactivating PI3K/AKT pathway (40). However, no report has investigated the role of the genetic variants of *INPP5D* in NSCLC survival. In the present study, for the first time, we found that the genetic variants of *INPP5D* were significantly associated with OS and DSS in NSCLC patients. The *INPP5D* rs13385922 T allele showed a significant unfavorable effect on NSCLC survival and an association with increased mRNA expression levels of *INPP5D*. However, the mRNA expression of *INPP5D* was not associated with NSCLC survival. We also found that the mRNA expression levels of *INPP5D* were down-regulated in LUSC but not in LUAD, suggesting tumor specificity between LUSC and LUAD. Moreover, the mRNA levels of *INPP5D* may also be possibly modulated by other factors, such as the regulation of RNA transcription and degradation. Nevertheless, the down-regulation of the *INPP5D* mRNA expression in NSCLC identified in the present study is in line with a previous study (40). Taken together, we concluded that *INPP5D* might be a potential tumor suppressor gene in NSCLC.

*EXOSC3* (also known as exosome component 3) is located on chromosome 9 and composed of 275 amino acids. *EXOSC3* is one of the constituent elements of RNA exosomes, and mutations in *EXOSC3* have been linked to pontocerebellar hypoplasia and spinal motor neuron degeneration (41). One study showed that exosc3-deficient B cells were impaired in the ability to undergo normal levels of somatic hypermutation and class switch recombination (42). Few studies have investigated the role of *EXOSC3* in cancer. A previous study showed that the protein expression of EXOSC3 was significantly up-regulated in pancreatic cancer tissue using protein-deep sequencing (43), but there were no published studies that explored the associations between genetic variants of *EXOSC3* and NSCLC survival. In the present study, our results suggest that the *EXOSC3* rs3208406 G allele may predict a reduced risk of survival for NSCLC patients and up-regulated the mRNA expression levels of *EXOSC3* in the 1000 Genomes Project and whole blood samples. Our findings on this SNP-mRNA correlation suggested that *EXOSC3* rs3208406 G may influence the prognosis of NSLCLC via modulating the mRNA expression of *EXOSC3*. However, functional experiments should be designed to explore the potential molecular mechanisms underlying the observed SNP-mRNA associations.

The observed SNP-survival associations in the present study suggested that genetic variants in the immunity B cell-related genes might be potential therapy targets for NSCLC. However, several methodological weaknesses in this study should be acknowledged. First, due to NSCLC patients in the two GWAS datasets were only limited to Caucasian descents, our findings may not be generalizable to other populations with different ethnicities. To address this gap, we will design replication study with other larger and independent populations from different races or geographic regions. Second, because the lack of detailed genotype data and clinical outcomes information in the HLCS study, we conducted the combined and stratified analyses with data only from the PLCO trial, and the results should be interpreted cautiously. Third, no further clinical information on nutrition status and details of treatment were available for further analysis. Moreover, although the two identified SNPs were identified to be associated with NSCLC survival, the potential mechanisms are not clear. Further experiments should be undertaken both *in vivo* and *in vitro* to better understand the mechanisms underlying the observed associations between two identified SNPs and NSCLC survival.

## Data availability statement

Publicly available datasets were analyzed in this study. This data can be found here: dbGaP accession numbers: phs000093.v2.P2 and phs000336.v1.p1.

## Ethics statement

The studies involving humans were approved by The PLCO trial was approved by the National Cancer Institute and the institutional review boards of each involved center. The use of the PLCO trial and HLCS study for experimentation was approved by the Internal Review Board of Duke University School of Medicine (Project #Pro00054575) and the dbGaP database (Project #6404). The studies were conducted in accordance with the local legislation and institutional requirements. Written informed consent for participation was not required from the participants or the participants’ legal guardians/next of kin in accordance with the national legislation and institutional requirements.

## Author contributions

GL: Conceptualization, Formal analysis, Funding acquisition, Methodology, Software, Writing – original draft. HL: Conceptualization, Data curation, Validation, Writing – original draft. HW: Formal analysis, Writing – original draft. XT: Formal analysis, Writing – original draft. SL: Methodology, Writing – review & editing. MD: Data curation, Formal analysis, Validation, Writing – original draft. DC: Funding acquisition, Methodology, Writing – review & editing. QW: Conceptualization, Funding acquisition, Methodology, Writing – review & editing.
