# Supplementary material for: Potentially functional variants of INPP5D and EXOSC3 in immunity B cell-related genes are associated with non-small cell lung cancer survival
Source: Front Immunol. 2024 Aug 8;15:1440454. doi: 10.3389/fimmu.2024.1440454 (PMC11338758; doi:10.3389/fimmu.2024.1440454)
Supplement: Supplementary file 2 [file DataSheet_1.doc]

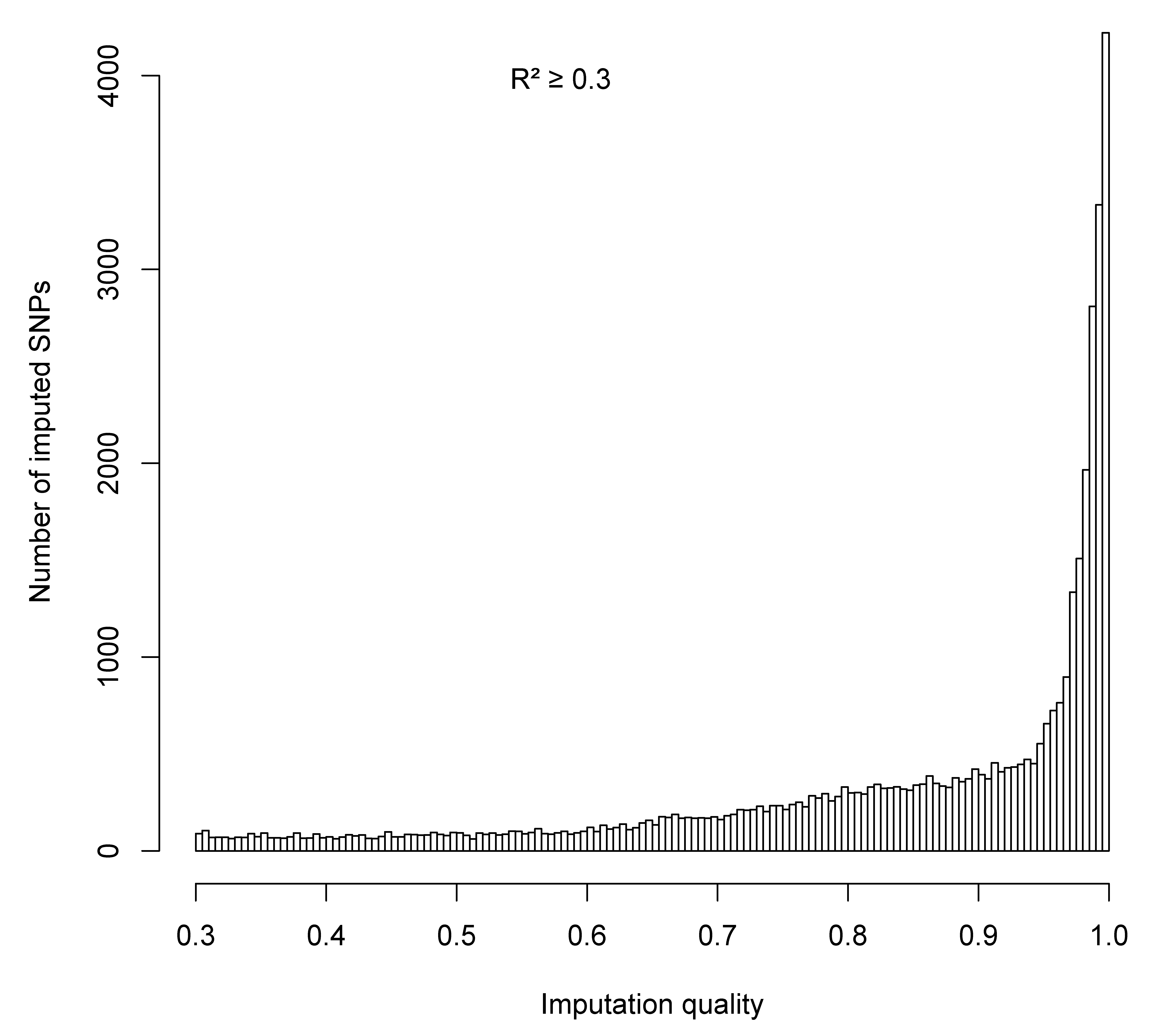


**Supplementary Figure 1:** The distribution of the imputation information score of the present study.


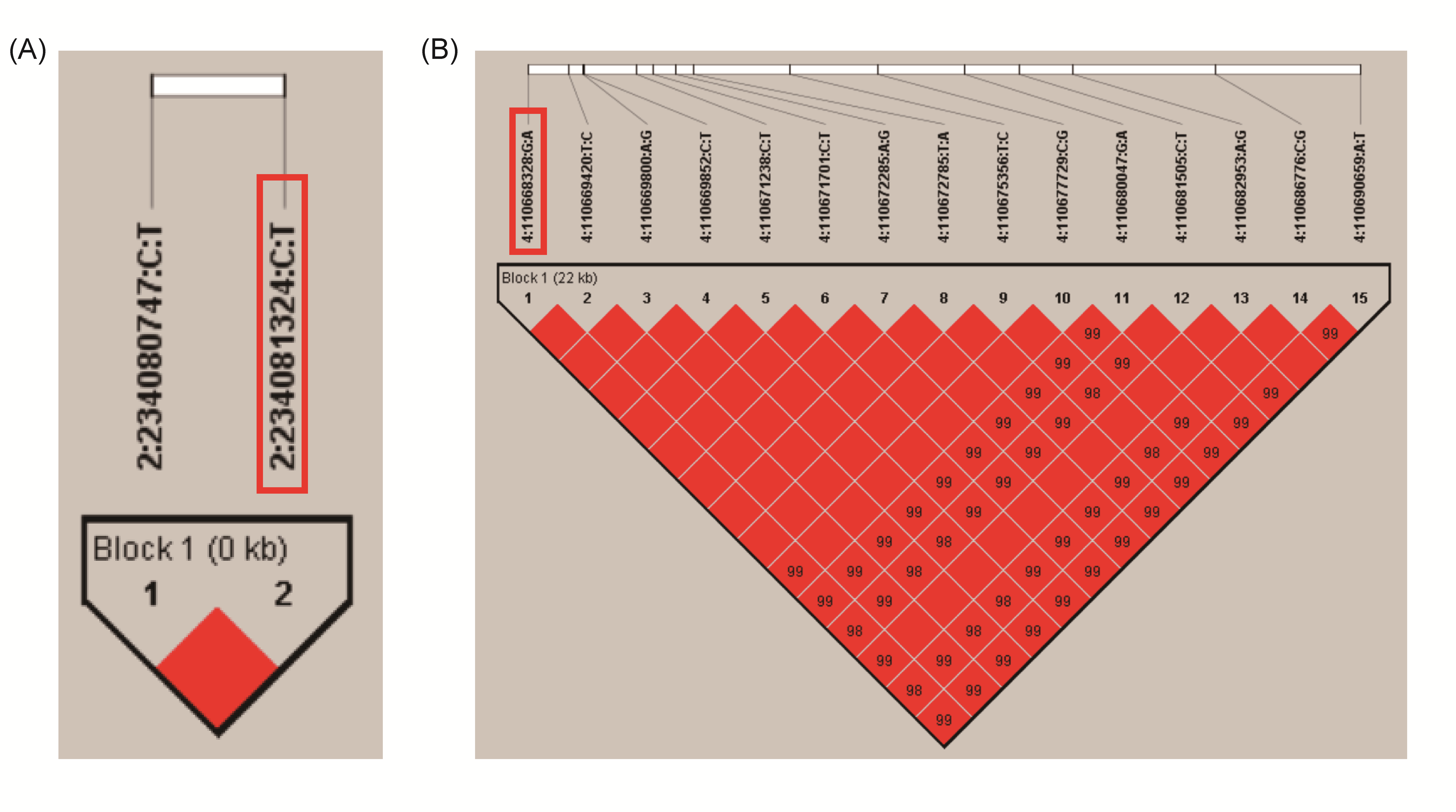


**Supplementary Figure 2:** Linkage disequilibrium (LD) analysis for 17 significant SNPs located in two genes. (A) two SNPs in *INPP5D*, (B) 15 SNPs in *CFI*.

Abbreviations: SNPs, single nucleotide polymorphism.


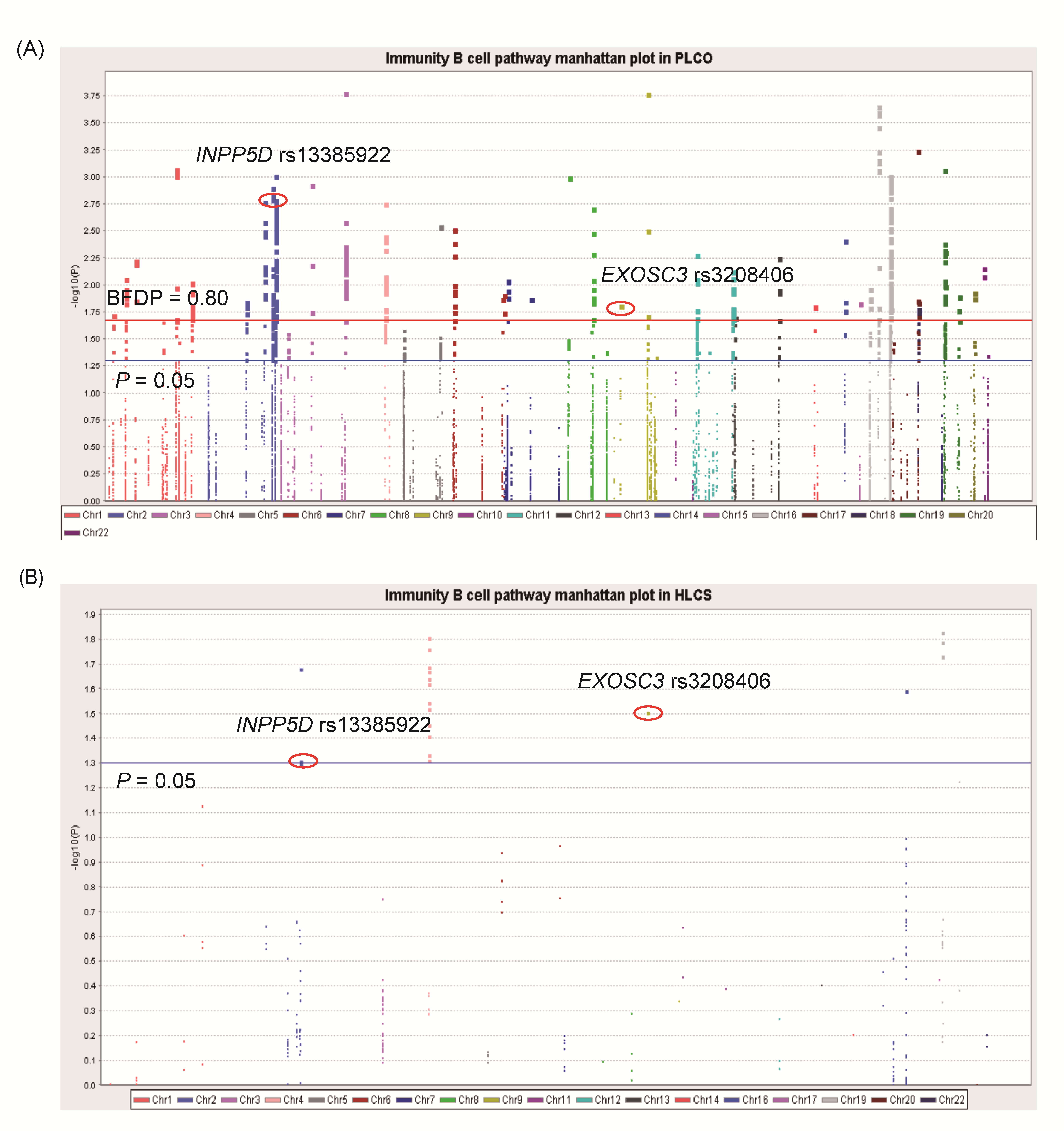


**Supplementary Figure 3.** Manhattan plot in the PLCO trial and HLCS study. (A) Manhattan plot for 10,776 SNPs of immunity B cell-related genes in the PLCO trial. (B) Manhattan plot for 369 SNPs of immunity B cell-related genes in the HLCS study. The blue horizontal line indicates *P* = 0.05 and the red line indicates BFDP = 0.80.


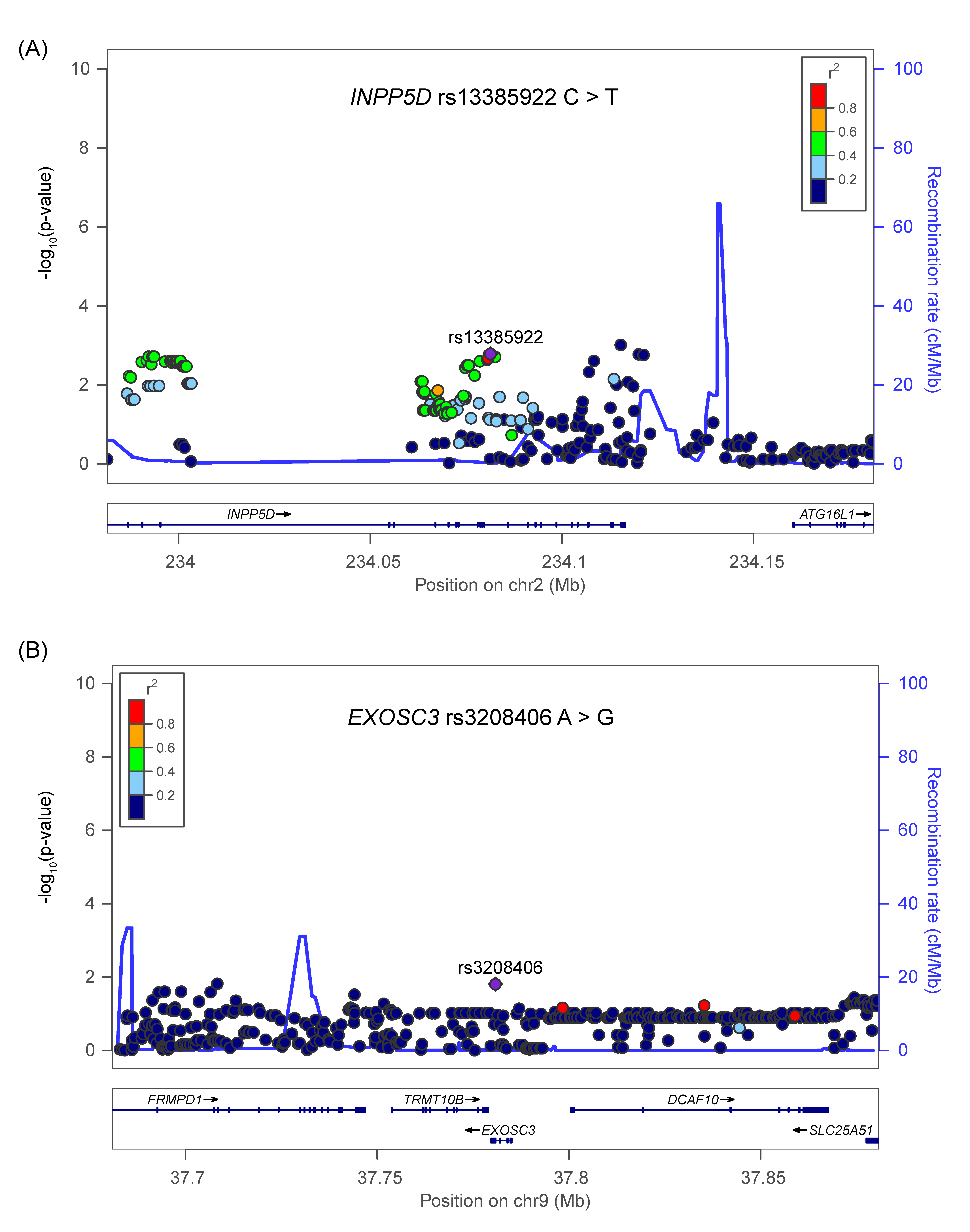


**Supplementary Figure 4.** Regional association plots for the two independent SNPs in the immunity B cell-related genes.

Regional association plots included 100kb up or downstream of (A) *INPP5D* and (B) *EXOSC3*. Data points are colored according to the level of linkage disequilibrium of each pair of SNPs based on the hg19/1000 Genomes European population. The left-hand y-axis shows the association *P*-value of individual SNPs in the discovery dataset, which is plotted as -log10 (P) against chromosomal base-pair position. The right-hand y-axis shows the recombination rate estimated from HapMap Data Rel 22/phase II European population.


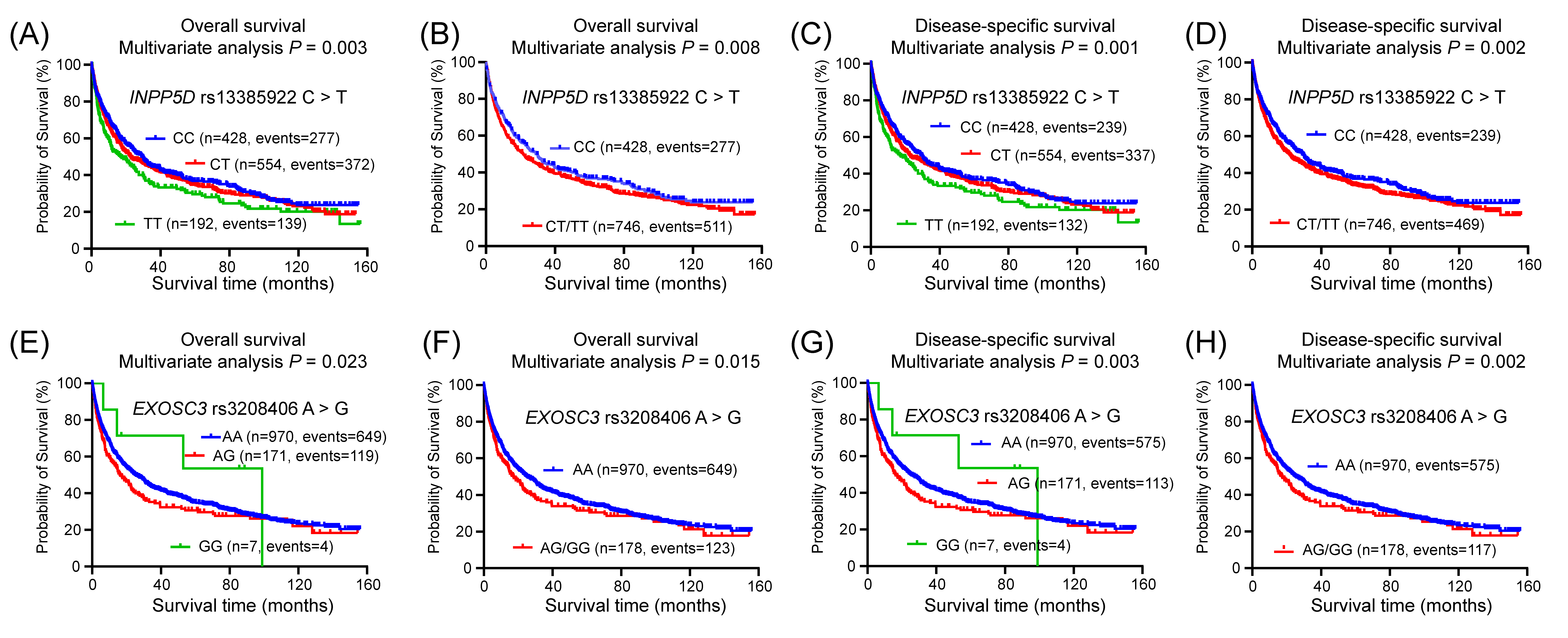


**Supplementary Figure 5**. Prediction of OS and DSS survival with genotypes of *INPP5D* rs13385922 and *EXOSC3* rs3208406 in the PLCO dataset. Kaplan–Meier survival curves of *INPP5D* rs13385922 for OS with (A) additive model, (B) dominant model; for DSS with (C) additive model, (D) dominant model; *EXOSC3* rs3208406 for OS with (E) additive model; (F) dominant model; for DSS with (G) additive model; (H) dominant model.


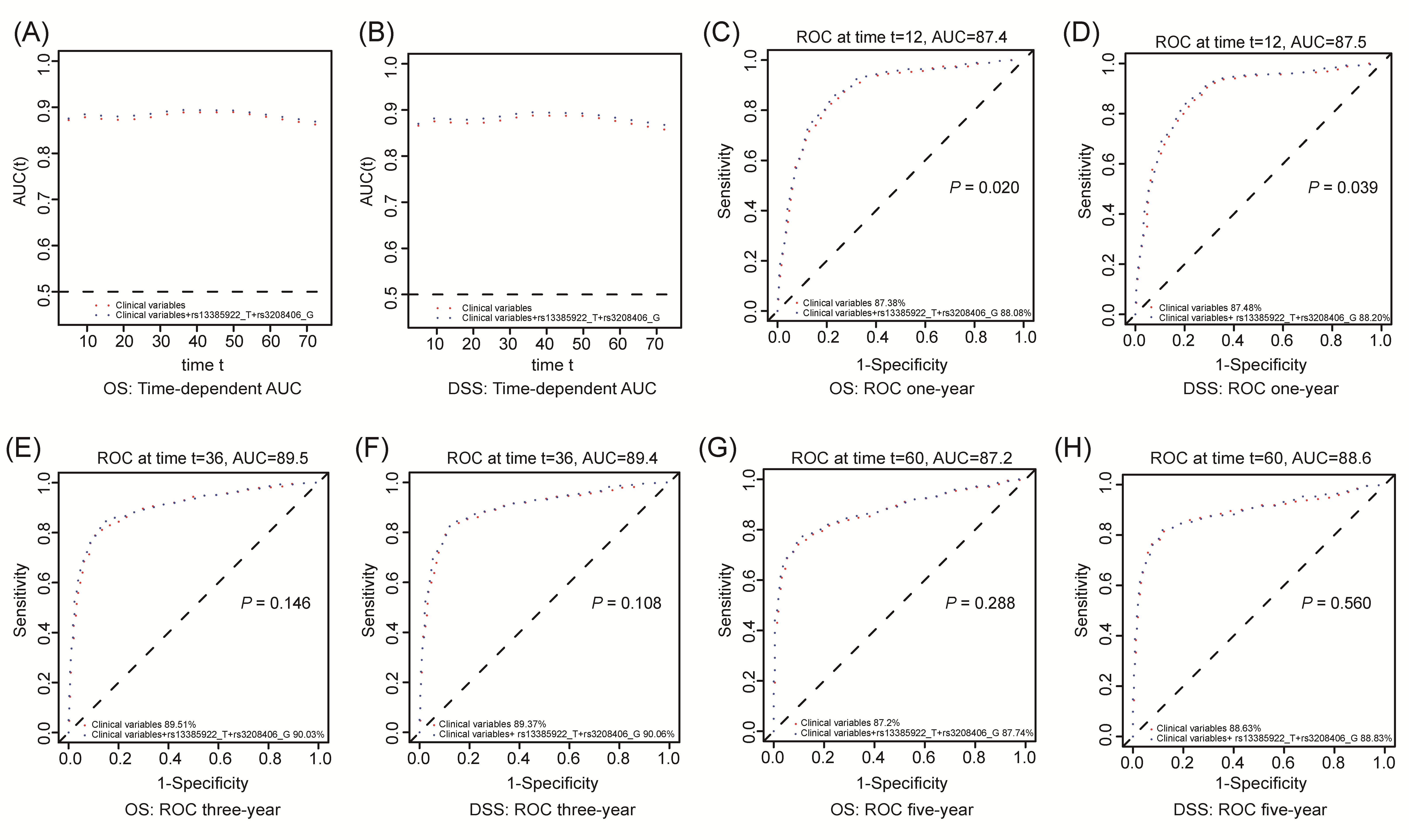


**Supplementary Figure 6.** NSCLC survival prediction with two SNPs by AUC and ROC curve at the 12^th^, 36^th^, and 60^th^ month. Time-dependent AUC analysis of (A) OS and B) DSS based on age, sex, smoking condition, histology, tumor stage, chemotherapy, surgery, principal component, and the two SNPs; The ROC curve evaluation for (C) OS and (D) DSS at 12^th^ month, (E) OS and (F) DSS at 36^th^ month, (G) OS and (H) DSS at 60^th^ month.


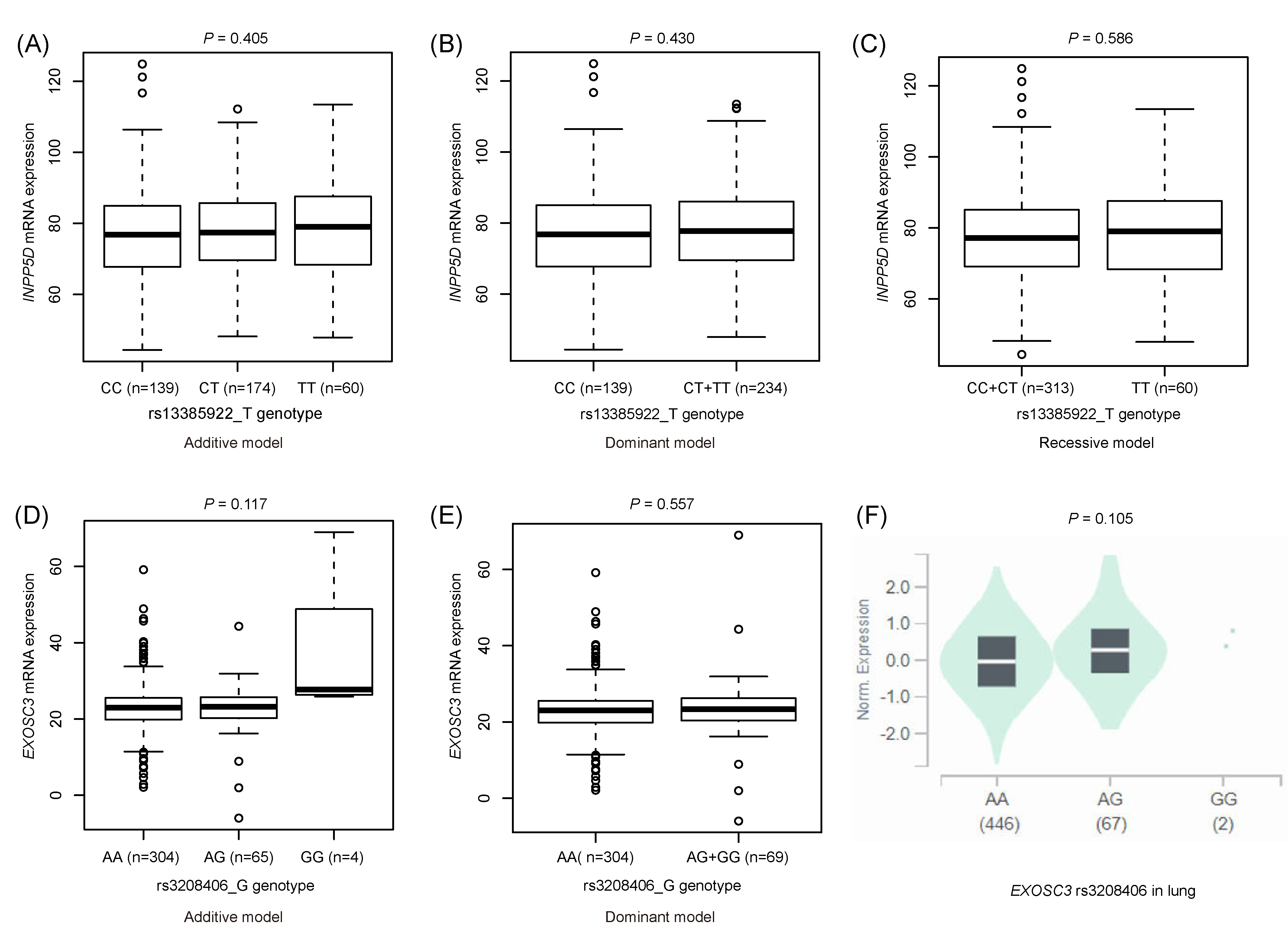


**Supplementary Figure 7.** Correlation between two genotypes and their corresponding mRNA expression levels.

The eQTL from 1000 Genomes project for *INPP5D* rs13385922 in (A) additive model, (B) dominant model, and (C) recessive model; for *EXOSC3* rs3208406 in (D) additive model and (E) dominant model; The eQTL from GTEx project for *EXOSC3* rs3208406 in (F) normal lung tissue.


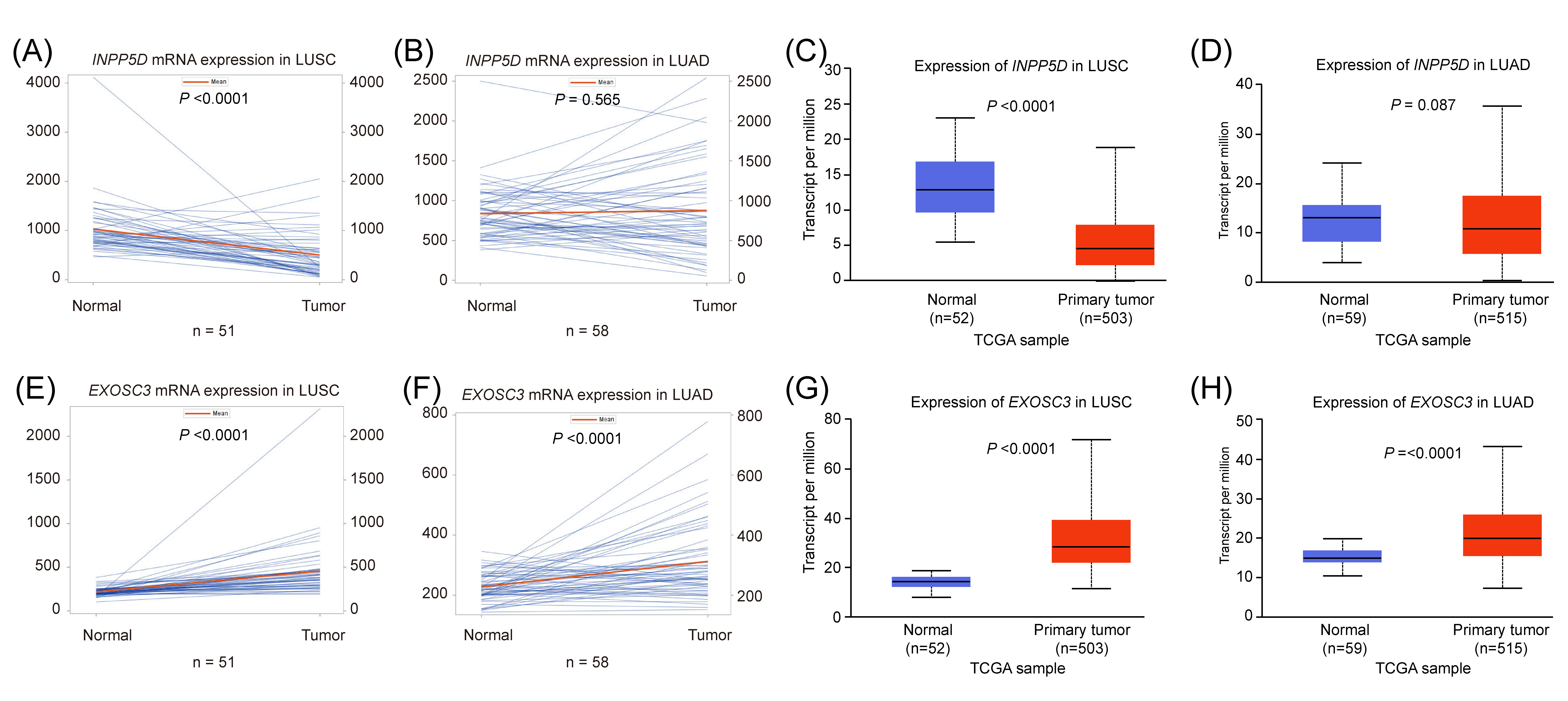


**Supplementary Figure 8**. Paired and non-paired mRNA expression analysis of *INPP5D* and *EXOSC3* in the TCGA database*.*

Both paired and non-paired analysis suggested that *INPP5D* mRNA expression level was significantly down-regulated in LUSC, but not in LUAD (A-D); *EXOSC3* mRNA expression level was up-regulated in LUSC and LUAD (E-H).
